# A new zygodactylid species indicates the persistence of stem passerines into the early Oligocene in North America

**DOI:** 10.1186/s12862-018-1319-6

**Published:** 2019-01-05

**Authors:** Tobin L. Hieronymus, David A. Waugh, Julia A. Clarke

**Affiliations:** 1 Department of Anatomy and Neurobiology, Northeast Ohio Medical University, 4209 State Rt 44, Rootstown, OH 44272 USA; 2 University of Texas at Austin, Jackson School of Geosciences, Austin, TX USA

**Keywords:** Passeriformes, Paleogene, Paleobiogeography, Zygodactylidae

## Abstract

**Background:**

The lake deposits of the informal Ruby Paper Shale unit, part of the Renova Formation of Montana, have yielded abundant plant fossils that document Late Eocene – Early Oligocene global cooling in western North America. A nearly complete small bird with feather impressions was recovered from this unit in in 1959, but has only been informally mentioned.

**Results:**

Here we describe this fossil and identify it as a new species of *Zygodactylus*, a stem lineage passerine with a zygodactyl foot. The new taxon shows morphological traits that are convergent on crown Passeriformes, including an elongate hallux, reduced body size, and a comparative shortening of proximal limb elements. The fossil documents the persistence of this lineage into the earliest Oligocene (~ 33 Ma) in North America. It is the latest occurring North American species of a group that persists in Europe until the Miocene.

**Conclusions:**

Eocene-Oligocene global cooling is known to have significantly remodeled both Palearctic and Nearctic mammal faunas but its impact on related avifaunas has remained poorly understood. The geographic and temporal range expansion provided by the new taxon together with avian other taxa with limited fossil records suggests a similar pattern of retraction in North America followed by Europe.

**Electronic supplementary material:**

The online version of this article (10.1186/s12862-018-1319-6) contains supplementary material, which is available to authorized users.

## Background

Zygodactylidae [1] is an extinct clade of small birds that possess a zygodactyl foot, with two pedal digits that are caudally deflected [2–6]. This clade includes the genera *Zygodactylus* [7], *Primozygodactylus* [8], *Eozygodactylus* [9], and *Primoscens* [10]. Described species are most abundant in Eocene deposits of North America and Europe, although they are also known from the Oligocene and early Miocene of Europe [2, 3, 7, 11]. Indeed, although parts of this clade were first described from the Miocene of Germany and France [7, 11], partial skeletons are only known from the early Oligocene and early Eocene of these same regions and the Eocene of North America. All younger material has been limited to isolated elements. Zygodactylids have been identified as allied with extant songbirds, or stem passerine taxa [3]. This recovered relationship and strong support for a passerine/parrot sister taxon relationship [12–14] opened up the possibility that a zygodactyl foot may be ancestral to the entire group [3].

The specimen described here was collected by Herman F. Becker and his field assistant Paul Roper in the last hours of their 1959 field season, while waiting for local rancher Elwyn Metzel to transport their packed camp and specimens to the train station [15]. The focus of Becker’s expedition was a floral study of Medicine Lodge and Ruby Basins in southwest Montana for the University of Michigan Museum of Paleontology. The bird fossil was transferred to the care of Glenn Jepsen at the Natural History Museum of Princeton, where it was prepared and put on display [16] but never formally described. The main slab of the specimen was figured in an overview of the Ruby Basin Flora [17] with suggested taxonomic affinity to kinglets or titmice based on the preserved feather crest. Its counterpart was figured in an overview of intermontane basin paleofloras, with suggested affinity to sandpipers [18]. In 1985, the specimen was transferred to the Yale Peabody Museum of Natural History. It was illustrated in an examination of fossilized melanosomes and identified as an undescribed Oligocene zygodactylid bird [19] based on a preliminary identification by one of us (Clarke). Here, we assess the phylogenetic affinities of the specimen and its implications for understanding the evolution of the North American Avifauna.

## Results

### Systematic paleontology

Aves Linneaus 1758

Parapasseres Mayr 2015

Zygodactylidae Brodkorb 1971

*Zygodactylus* Ballmann 1969

*Zygodactylus ochlurus* sp. nov.

### Holotype

**Fig. 1:**
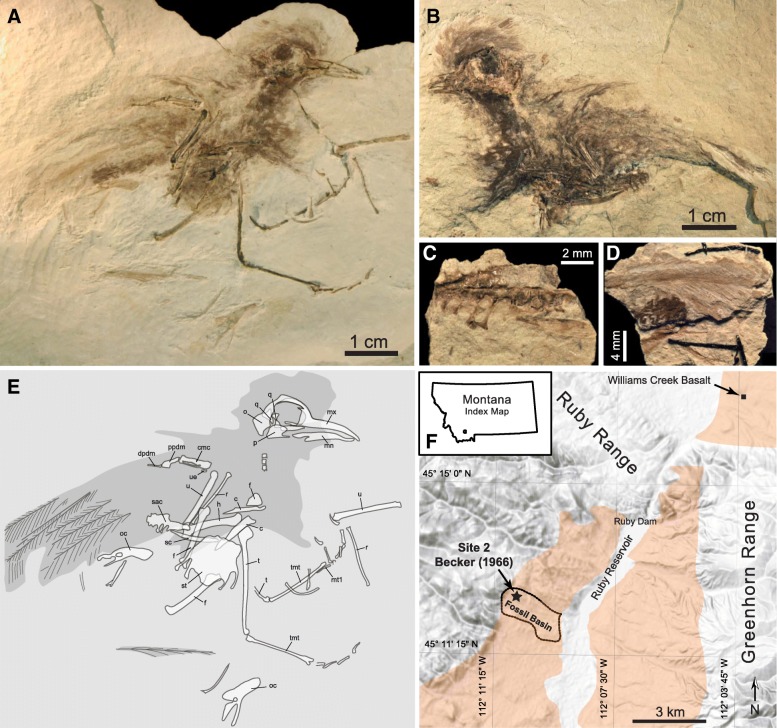
Holotype specimen of *Zygodactylus ochlurus* n.sp. (YPM VPPU 17053) with interpretive drawing and geologic context. **a** Main slab and **b** counterpart of holotype, with **c-d** two interstitial fragments. **e** Line drawing with outlines from part, counterpart, and fragments superimposed. Abbreviations: *c*, coracoid; *cmc*, carpometacarpus; *dpdm*, distal phalanx digiti majoris; *f*, femur; *h*, humerus; *mn*, mandible; *mt1*, first metatarsal; *mx*, maxillary rostrum; *o*, occiput; *oc*, os coxa; *of*, omal extremity of furcula; *p*, palatine fragment; *ppdm*, proximal phalanx digit majoris; *q*, quadrate; *r*, radius; *sac*, synsacrum; *sc*, scapula; *st*, sternum; *t*, tibiotarsus; *tmt*, tarsometatarsus; *u*, ulna; *ue*, ulnare. **f** Simplified geologic map of the Upper Ruby River Basin. Inset shows location of the map in Southwestern Montana. Orange overlay indicates surface extent of Renova Fm [61]

YPM VPPU 17053, partially articulated skeleton preserved on main slab and counterpart, including two smaller flakes detached from the counterpart (Fig. 1a-e). Feather impressions surround the skeleton. Most elements are preserved as mouldic impressions and fragments of bone.

### Etymology

From ὀχληρός (*ochlhros*), Classical Greek for troublesome, in reference to “Trouble,” an orphaned magpie chick that Becker hand-reared in the Fossil Basin camp during his 1959 field season [17].

### Type locality and horizon

Becker locality #2 in Fossil Basin, Upper Ruby Valley, Madison County [15, 20, 21] (Fig. 1f). Plant fossils from this locality are assigned to the Ruby Basin Flora, lithostratigraphically positioned at the boundary between the Climbing Arrow and Dunbar Creek Members of the Renova Formation [22]. The Dunbar Creek Member is locally capped by the Williams Creek basalt, K-Ar dated to 32.2 ± 0.4 Ma [23], providing a hard upper bound for the age range. Paleoclimate studies place the Ruby Flora in the earliest Oligocene, ca. 33 Ma [22].

### Diagnosis

*Zygodactylus ochlurus* shares the combination of a large intermetacarpal process, a dentiform process of the carpometacarpus, a zygodactyl foot, and a greatly elongate tarsometatarsus (longer than or subequal to the humerus) unique to Zygodactylidae [5, 6, 22]. The new taxon is assigned to *Zygodactylus* based on the distal projection of metacarpal III past metacarpal II, and on the presence of a convexity proximal to the trochlea of metatarsal IV [2, 6]. The latter character is automorphic for *Zygodactylus*.

*Z. ochlurus* is differentiated from all other *Zygodactylus* spp. by (1) anterior projection of cranial cnemial crest greater than anteroposterior width of tarsometatarsus, (2) hallucal digit proximal phalanx longer than pedal digit III proximal phalanx, and (3) diminutive size: most other limb elements are approximately two-thirds the size of corresponding elements in congenerics *Zygodactylus grivensis*, *Z. ignotus*, *Z. luberonensis*, and *Z. grandei* (Table 1) [2, 6, 7, 11]. Complete fusion of the synsacral vertebrae in YPM VPPU 17053 are taken here as an indicator of skeletal maturity, and thus adult size, for this specimen.

**Table 1:** Selected measurements of *Zygodactylus ochlurus* n.sp. compared to other zygodactylids [3–6, 8, 9]

	SKL	COR	HUM	ULN	CMC	FEM	TIB	TMT	pI1	pI2	pII1	pII2	pII3	pIII1	pIII2	pIII3	pIII4	GM	FEM/ GM	CMC/ GM	pI1/ pes	pIII1/ pes
YPM VPPU 17053 *Zygodactylus ochlurus* n.sp. Holotype	21.0	~ 8.7/ ~ 8.7	12.5/	~ 13.2/ 14.7	6.7/	/ 10.3	/ ~ 21.4	15.6/ 15.4	3.8/	2.0/	3.2/	3.0/	3.3/	/ 2.8	/ 3.3	/ 3.0	/ 2.0	12.6	0.82	0.53	0.23	0.17
SMF Av 519^a^ *Zygodactylus luberonensis* Holotype	35.0	14.7/	17.2/ 17.2	18.1/	/ 8.6	~ 19.5/	34.8/ 34.7	24.6/ 24.5	/ 4.6	/ 2.1	/ 6.5	/ 4.6	/ 2.7	/ 7.1	/ 6.5	/ 5.4	/ 3.1	18.8	1.04	0.46	0.16	0.25
FMNH PA 726^b^ *Zygodactylus grandei* Holotype	21.1	13.7/	18.3/18.6	19.3/	~ 8.5	15.1/	33.0/33.6	21.4/20.4	4.6/4.3	2.6/2.7	/5.2	4.7/4.6	3.0	6.0/5.8	5.0/5.2	4.2/4.4	3.8/3.9	17.9	0.84	0.47	0.17	0.22
USNM 299821^c^ *Eozygodactylus americanus*	~ 19		16.8/ 16.8	~ 19/ 19.1	/ 8.8																	
WDC-CGR-014^c^ *Eozygodactylus americanus*		13.8/ ~ 13.1	/ ~ 17.2	~ 18.2/	8.8/	19.7/	/ 30.6	21.7/ 21.8	5.4/ ~ 5.6	2.5/ ~ 2.0	/ 5.9	5.1/ 5.2	2.9/ ~ 2.2	~ 6.0/ ~ 4.6	5.7/ 5.7	4.7/ 4.8	~ 2.7/ 3.3	18.2	1.08	0.48	0.21	0.20
SMF-ME 1758a + b^d^ *Primozygodactylus major* Holotype			~ 28.4/	~ 31.1/	~ 12.0/	24.6/	39.0/ 39.8	28.0/										25.7	0.96	0.47		
SMF-ME 2108^d^ *Primozygodactylus ballmanni* Holotype			21.0/ 20.5	22.9/ 22.9	~ 9.0 /~ 9.5	20.8/	33.0/	24.6/										20.5	1.02	0.45		
SMF-ME 2522a + b^d^ *Primozygodactylus danielsi* Holotype			16.5/ 16.4	18.3/ ~ 17.4	8.2/ 8.1	/ 16.5	27.4/ 27.3	19.6/ ~ 18.3	4.0/	2.4/	4.8/	4.3/	2.4/	5.7/	4.9/	4.3/	2.6/	16.5	1.00	0.49	0.17	0.24
SMF-ME 1074^d^ *Primozygodactylus eunjooae* Holotype						17.5/	29.8/	~ 21.7 /~ 21.7														
SMF-ME 11091a + b^d^ *Primozygodactylus quintus* Holotype			19.7/ 20.0	20.7/20.7	9.5/9.5		32.5/32.5	22.8/23.1														
SMF-ME 11171a + b^d^ *Primozygodactylus longibrachium* Holotype			> 19/ 19.6	21.4/> 21	9.6/		29.5/29.5	/19.0														

^a^after [2]

^b^after [6]

^c^after [9]

^d^after [5]

*SKL* Rostrocaudal skull length, *COR* Coracoid, *HUM* humerus, *ULN* ulna, *CMC* carpometacarpus, *FEM* femur, *TIB* tibia, *TMT* tarsometatarsus, *pI1 – pIII4* pedal digit (I-III) phalanx (1–4), measured in mm. *GM* geometric mean of HUM, ULN, CMC, FEM, TIB, and TMT; pI1/pes and pIII1/pes: pedal digit I/III proximal phalanx, scaled against the sum of lengths of the non-ungual phalanges of pedal digits I & III

*Z. ochlurus* is differentiated from *Eozygodactylus americanus* by (1) shorter lateral sternal trabeculae, (2) femur shorter than humerus, (3) ulna approximately equal to tarsometatarsus in length, (4) presence of a dentiform process on the cranial margin of the carpometacarpus.

*Z. ochlurus* is differentiated from all *Primozygodactylus* spp. by (1) a large dorsal supracondylar process of humerus, (2) femur shorter than humerus, (3) metacarpal III longer than metacarpal II, and (4) obturator foramen continuous with ischiopubic fenestra. The new taxon is differentiated from all *Primozygodactylus* spp. except *P. danielsi* by a relatively elongate proximal phalanx of pedal digit II.

### Measurements

See Table 1.

### Description

#### Skull

The skull is badly crushed, and is split between main slab (Fig. 1a) and counterpart (Fig. 1b) on a largely parasagittal plane through the right orbit. A short maxillary rostrum relative to skull length is more similar to *Eozygodactylus americanus* than *Z. luberonensis* [3, 9]. The bodies and otic processes of both left and right quadrate are visible on the part. Dark-stained matrix in the middle of the skull rests in the right orbit, and obscures the orbital process of the right quadrate. The occipital region remains on the counterpart, including the outline of the left paroccipital process and the middle ear cavity (cavum tympanicum). The outline of the tympanic crest is similar to *E. americanus*. The mandibular rami are distorted, but appear to display a ventral curvature similar to *E. americanus*. Crushed remnants of the left palatine are visible rostral to the middle ear cavity on the counterpart.

#### Vertebral column

Faint remnants of the cervical column are split between main slab and counterpart, without substantial exposed surfaces. The body of the sacrum is split between counterpart and first intermediate flake (Fig. 1c). It appears to contain ~ 11 vertebrae; nine are visible, but the series is cranially incomplete. Costal processes of the acetabular vertebra are visible on both slabs, while the adjacent body of the sacrum is largely crushed onto the counterpart. Foramina intertransversaria look to be enclosed for at least two of the caudal sacral series.

#### Pectoral girdle and limb

The sternal keel is preserved in the main slab. The body of the sternum forms a mouldic impression around the fragmented keel. Spina externa may be fragmentarily preserved alongside the left coracoid on the part, but its outline cannot be determined with any certainty. Right cranial portion of sternum, showing the pila coracoideus, is preserved on the counterpart. There are four sternal incisions caudally, with medial and lateral trabeculae ending in approximately the same plane as the broad caudal midline extremity.

Left scapula is preserved on the counterpart, with matching impression in first intermediate flake. The body of the scapula is less curved than in *E. americanus*.

An impression of the right coracoid is exposed in dorsal view on the part, missing medial and proximal parts of the flange. The impression shows a weakly developed procoracoid process, as in *Z. luberonensis*. An incompletely exposed bony remnant adjacent to the right coracoid is tentatively identified as the triangular omal extremity of the furcula. Crushed portions of the left coracoid, without the flange, are preserved in dorsal view on the part. The cranial end projects beneath the impression of the sternum and ends in close proximity to the cranial end of the left scapula.

The left humerus is preserved as an impression of the cranial surface of the shaft with crushed portions of the head on the main slab, and as fragmentary remains on the counterpart. The humerus is stocky and slightly shorter than the ulna. The deltopectoral crest morphology is poorly defined as a faint impression as the element lacks most of its proximodorsal margin. The humeral head is more dorsoventrally narrow than in *Z. luberonensis*. An impression of a prominent dorsal supracondylar process is apparent proximal to the impression of the dorsal condyle. A narrow flexor process protrudes distal to the ventral condyle, and a portion of this structure is also visible in the first intermediate flake. Humerotricipital and scapulotricipital sulci are present.

The left ulna is preserved as an impression on the main slab, with fragmentary remnants of the body on the counterpart. The distal end and body of the right ulna are preserved on the main slab, with a small fragment of the proximal end preserved on the counterpart. Papillae remigales are not apparent, and the ulnar shaft is straight. The olecranon process is poorly preserved, with a moderate point, similar to *Z. luberonensis* but less pronounced than in extant Passeriformes.

Left radius is preserved as a thin impression on the main slab and as fragments on counterpart, positioned slightly out of articulation with the left ulna. Right radius is preserved as collapsed fragments in the main slab, fully disarticulated from the right ulna. The left ulnare is a poorly-exposed and crushed body on the main slab. The radiale is not visible.

**Fig. 2:**
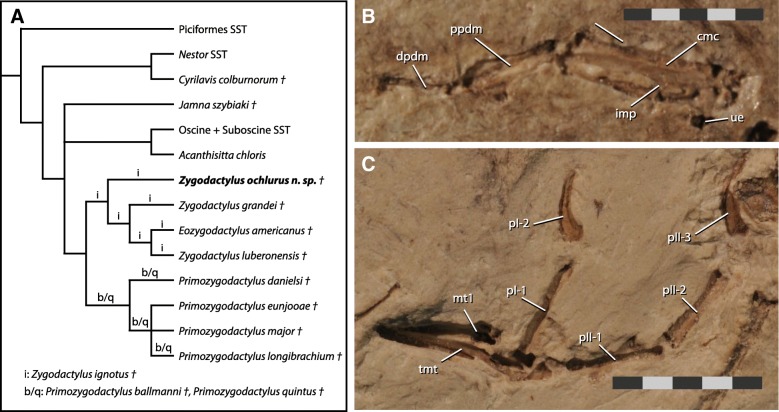
Phylogenetic context of *Zygodactylus ochlurus* n.sp. **a** Reduced consensus of the 30 most parsimonious trees from phylogenetic analysis. Positions of variable taxa are indicated above the branches they occupy in the full tree set. **b** Left manus showing intermetacarpal process (imp) **c** Left pes showing elongate proximal phalanx of digit I (pI-1)

Left carpometacarpus is preserved as a crushed body and impression on the main slab. Metacarpal I is broken off but the area of breakage on metacarpal II suggests it would have been quite short. The ulnocarpal trochlea is preserved as a faint impression and does not appear to be large or significantly projected caudoventrally. Metacarpal III extends distal to metacarpal II. A prominent intermetacarpal process is visible within the narrow intermetacarpal space (Fig. 2b). A dentiform process is visible on the cranial margin of major metacarpal. Left major digit is preserved in articulation with carpometacarpus on the main slab. All digits are preserved as impressions in the main slab. Digit III:1 appears to be displaced to lie along side distal II:1. Phalanx II:1 flares distally and is partially overlain by digit II:2. As preserved, the impression of digit II:1 appears to possibly bear a distinct caudodistal processus not dissimilar to that seem in some Galbulae. However, the impression shows a proximodistal furrow that is interpreted as more consistent with an underlying, separate, element such as a carpal or phalanx.

#### Pelvic girdle and limb

Left and right pelvic elements are disarticulated from the sacrum and preserved on the main slab, with an additional impression of left pelvic elements on the second intermediate flake (Fig. 1d). The ilium is short and narrow with its preacetabular portion broadly recurved dorsally and longer than its postacetabular portion. The pubis is thin and angles away from the ischium rather than being subparallel to its ventral margin. The obturator foramen is continuous with the ischiopubic fenestra. The right femur is preserved as an impression on main slab, with the distal end passing ventral to sternal impression. Left femur crosses under the impression of the left humerus on the same slab. The femora (Fig. 1e) are elongate and straight. The trochanteric crests are not well projected proximally.

The body and medial cnemial crest of the right tibiotarsus are preserved as an impression with collapsed fragments on the main slab. The large cranial cnemial crest, visible as an impression on the counterpart, is strongly projected cranially and dorsally. Distal end of the left tibiotarsus is visible on the main slab; body may continue beneath the sternum. Both tarsometatarsi are partially crushed. The right tarsometatarsus shows a prominent lateral plantar crest of the hypotarsus, as well as a slight convexity proximal to trochlea IV. Left metatarsal I visible as a separate element alongside the left tarsometatarsus. Pedal phalanges of left digits I, II, and III, right digit III preserved in articulation on the main slab. Digit I:1 is proportionally more elongate in the new taxon than in *Z. luberonensis* and *P. danielsi* (Table 1), but comparable to the proportional lengths of digit I:1 in basal Psittacopasseres *Avolatavis tenens* [24] and *Messelastur gratulator* [25].

#### Feathers

Capital and cervical tract feathers preserved as a darker halo of filamentous structures. Distal primary remiges (IV-IX?) are preserved as impressions on the main slab and second intermediate flake.

## Discussion

### Phylogenetic hypotheses

Analysis with the complete set of 39 equally weighted characters resulted in 30 most parsimonious trees, from implicit enumeration in TNT v1.5. IterPCR identified *Zygodactylus ignotus*, *Primozygodactylus ballmanni*, and *Primozygodacylus quintus* as unstable taxa. A reduced consensus of these 30 trees (Fig. 2a) recovers Zygodactylidae as a monophyletic clade, with further separation of *Primozygodactylus* and (*Zygodactylus* + *Eozygodactylus*) into stable clades recovered in all trees. *Z. ochlurus* is recovered as the sister taxon of all other *Zygodactylus* species.

### Morphological diversity within Zygodactylidae

The overall size and limb proportions of *Z. ochlurus* stand out among zygodactylids. When compared using the geometric mean of major limb element lengths (humerus, ulna, carpometacarpus, femur, tibiotarsus, and tarsometatarsus) as a proxy for size, *Z. ochlurus* is ~ 25% smaller than any other zygodactylid, and only half the size *Primozygodactylus major*, the largest member of the family. When individual element lengths are scaled to this body size proxy, *Z. ochlurus* shows a distinctive combination of relatively short femur and relatively long carpometacarpus (Table 1).

Pedal phalanx proportions are another area where *Z. ochlurus* expands the morphological diversity seen in zygodactylids. The elongate proximal phalanx of the hallux that is diagnostic of *Z. ochlurus* is convergent with pedal morphology in oscine and suboscine passeriforms. The proportionally longer pedal digits seen in *Z. luberonensis* have been proposed to suggest this species was perhaps more terrestrial and suited to more open environments [3]. While the morphology of the new species indicates it is a part of the same zygodactylid subclade, the proportions of the pedal phalanges relative to each other and the tarsometatarsus in *Z. ochlurus* are more similar to those seen in *Primozygodactylus* spp., and are more consistent with arboreal habit [26]. Taken together, the limb proportions of *Z. ochlurus* suggest greater ecological diversity within the *Zygodactylus* clade than has previously been appreciated.

### Palaeoecological implications

Paleoclimate interpretations from the Ruby paleoflora show Fossil Basin as a seasonally arid, high-altitude (~ 2500 m) temperate scrubland at the beginning of the Oligocene [22]. This setting is a strong contrast from the tropical-subtropical lowland settings in which other Z*ygodactylus* species have been preserved [27], and suggests that zygodactylids were not restricted to relictual tropical habitats following Late Eocene global cooling. Zygodactylid extinction may be expected to be explained by more complex factors. If their declining distribution tracked tropical-subtropical environments they may be expected to persist in parts of Africa and Asia [28].

Three major themes of passerine paleobiogeography have emerged from prior analyses. The first is the proposed persistence of the passeriform stem in the separated remnants of Gondwana in the Cenozoic [29]. Second is the vicariance and diversification of suboscines in South America, with subsequent radiation to Africa and Asia [30, 31]. Third is the vicariance, diversification, and radiation of oscines from Australia [32]. The geographic distribution of stem passeriform fossils is largely incongruent with the hypothesis of a persistent Gondwanan stem [28, 33]. *Z. ochlurus* supports a persistent Holarctic distribution of stem passerines from the Paleogene into at least the Early Oligocene of North America as well as Europe incongruent with a stem lineage restricted to the separated remnants of Gondwana.

The Eocene – Oligocene paleogeographic records of birds and mammals in Europe and North America show a consistent pattern. Early Eocene avian and mammalian faunas are similar between Europe and North America [34, 35]. Late Eocene faunas show a combination of similarity and endemic radiations [34, 36]. By contrast, the Eocene/Oligocene transition is marked by diverging patterns of extinction and faunal turnover. The geologic and paleofloral record in North America suggests a transition to seasonally arid climates 37–35 Ma [37], well in advance of similar transitions in Europe at 34–32 Ma. Clades with considerable species richness in the Eocene disappear from the North American record close to the Eocene/Oligocene boundary, (Coliiformes, mousebirds [38]; Todidae, todies [39]) or are not known after the middle Eocene (e.g., Podargiidae, frogmouths; and Coracii, rollers [40, 41]; Paleognathae [42, 43]) [34].

The disappearances of these taxa with widespread extant distributions have been proposed to track the retraction of key forested environments out of North America [40], similar to proposed patterns in primates [35]. The new species shows at least one subclade of zygodactylids, *Zygodactylus*, persists at least into the earliest Oligocene of North America. It is also this subclade that is known from the Oligocene and Miocene of Europe [2, 7, 11].

The European zygodactylid *Z. luberonensis* was a contemporary of the oldest known European suboscine, both recovered from the Luberon area in France, from deposits dated to ~ 30 Ma [44], ~ 6 Ma prior to the closure of the Tethys seaway. Additional passeriform taxa that cannot be confidently assigned within Eupasseres are known from the Rupelian Carpathian Basin of Poland (32–28 Ma) and the Frauenweiler deposits in Germany [45–50]. The earliest European record of oscines (songbirds), which comprise nearly half of the species-level diversity of all extant birds, comes from the late Oligocene deposits of Herrlingen in Germany, and dates to ~ 24 Ma [51]. The latter date coincides with the uplift of Wallacea in the southwestern Pacific, which is thought to have facilitated the dispersal of oscines from Australia [32]. With Miocene records from Grive-Saint-Alban and Wintershof, zygodactylids persisted in Europe for at least another 6–8 Ma after oscines arrived, at least until the Miocene climatic optimum [7, 11]. The oldest definitive crown passeriform currently known from North America is the Miocene *Miocitta galbreathi* (~ 16–13 Ma [52]). An older “passerine” from Florissant [53] is not diagnosable as a member of the crown, and may well represent a zygodactylid.

The early Oligocene may be characterized by Holarctic persistence of an increasingly diverse group of zygodactylids known from distinct environments including inferred arid, higher altitude sites in North America. During this time oscine and suboscine crown Passeres are inferred to be dispersing into these environments [51]. Competitive interactions, documented among extant passeriform groups, have been suggested as factors in the ecological divergence of the crown passeriform radiation [30]. New records are needed to elucidate dynamics and potential drivers of survivorship and extinction during this key interval. With similar body sizes, stem passerines occupy a range of ecological settings in the face of rapidly changing environments in the early days of an late Paleogene icehouse world.

## Conclusions

The morphology of the new taxon is convergent on crown Passeriformes in several respects. The new taxon has a smaller overall size than other zygodactylids. The limb proportions of the new taxon are also convergent on the ranges seen in crown Passeriformes, most notable in the length of proximal phalanx of the hallux.

Oligocene zygodactylids were not confined to relict tropical forests after Late Eocene global cooling. Paleoclimatic reconstructions place *Z. ochlurus* in temperate mountain scrubland, expanding the ecological range of Zygodactylidae.

The new taxon extends the temporal range of North American zygodactylids from the Early Eocene in to the Early Oligocene. This extension supports the idea that passerine stem taxa maintained a persistent presence in the Holarctic, from at least the time of the earliest estimated divergences within the crown (~ 52 Ma) until well after the proposed dispersal of oscine songbirds into the Holarctic from Australia (~ 16 Ma).

## Methods

### Taxon and character sampling & phylogenetic analysis

Anatomical features of the Ruby Basin specimen (YPM VPPU 17053) were assessed by comparison to specimens representing the majority of known species in Zygodactylidae: *Zygodactylus luberonensis* (SMF Av 519); *Zygodactylus ignotus* (BSP 18164); *Zygodactylus grivensis* (FSL 151); *Zygodactylus grandei* (FMNH PA 726); *Eozygodactylus americanus* (USNM 299821); *Primozygodactylus eunjooae* (SMF-ME 1074); *Primozygodactylus ballmanni* (SMF-ME 1768); *Primozygodactylus danielsi* (SMF-ME 2522); *Primozygodactylus longibrachium* (SMF-ME 11171); *Primozygodactylus major* (SMF-ME 2108); and *Primozygodactylus quintus* (SMF-ME 11091). Thirty-nine discrete morphological characters (Additional file 1: Appendix I) were coded for the new taxon, drawn largely from prior published analyses [2, 4, 6, 9, 24, 54–56]. Morphological characters of *Zygodactylus luberonensis*, *Zygodactylus grandei*, *Zygodactylus ignotus*, and *Primozygodacylus* spp. were evaluated from published sources [2, 3, 5, 6, 8, 11].

Character states for several outgroup taxa were included from published sources. Extant Passeriformes were represented by two terminal taxa: the basal passeriform *Acanthisitta chloris*, and a supraspecific terminal (SST) taxon representing basal states for oscine and suboscine passeriforms, using states reported from published studies [6] derived from a composite of *Tyrannus tyrannus*, *Thamnophilus caerulescens*, *Corvus brachrhynchos*, and *Menura novaehollandiae*. The putative stem passeriform *Jamna szybiaki* [45] was also included. Psittaciformes were represented by an SST containing *Nestor meridionalis* and *Nestor notabilis* [6, 57], as well as a terminal for the fossil stem psittaciform *Cyrilavis colburnorum* [57]. Piciformes were represented by an SST derived from *Dryocopus pileatus*, *Colaptes auratus*, *Galbula ruficauda*, and *Chelidoptera tenebrosa* [6].

Phylogenetic analysis used equal weights and a maximum parsimony estimator in TNT 1.5 [58, 59], with a search by implicit enumeration (branch and bound). *Z. grivensis* was excluded due to a large proportions of missing character data. The piciform SST was selected as an outgroup to root the resulting trees. Iterative Reduced Positional Congruence (iterPCR [60]) was used to identify unstable taxa in the set of most parsimonious trees.

## Additional file

Additional file 1: Appendix I Morphological character state descriptions. (DOCX 34 kb)

## Abbreviations

BSP: Bayerische Staatssammlung für Paläontologie und Historische Geologie, Munchen, Germany
FMNH: Field Museum of Natural History, Chicago, Illinois, USA
FSL: Faculté des Sciences de Lyon, France
SMF: Forschunginstitut Senckenberg, Frankfurt am Main, Germany
USNM: National Museum of Natural History, Smithsonian Institution, Washington, DC, USA
YPM: Yale Peabody Museum, New Haven, Connecticut, USA
