# Supplementary material for: A new zygodactylid species indicates the persistence of stem passerines into the early Oligocene in North America
Source: BMC Evol Biol. 2019 Jan 5;19:3. doi: 10.1186/s12862-018-1319-6 (PMC6321701; doi:10.1186/s12862-018-1319-6)
Supplement: Supplementary file 1 — Appendix I Morphological character state descriptions. (DOCX 34 kb) [file 12862_2018_1319_MOESM1_ESM.docx]

A new zygodactylid species indicates the persistence of stem passerines into the Early Oligocene in North America

Tobin L. Hieronymus, David A. Waugh, Julia A. Clarke

**Additional File 1**

# Appendix: Morphological character state descriptions

- 1. Skull: narial opening approximately rectangular (0); ovoid (1). Character 1 of Smith et al. (2018)
  2. Skull: narial opening: greater than 50% the length of the rostrum (0); equal to or less than 50% the length of the rostrum (1). Character 2 of Smith et al. (2018)
  3. Skull: jugal: bowed ventrally (0); straight or concave (1). Character 3 of Smith et al. (2018)
  4. Furcula, omal extremity: reduced, outline linear (0); wide, subtriangular omal extremity (1). Character 9 of Smith et al. (2018) and Mayr (2004); character 12 of Mayr (2015).
  5. Scapula: acromion, size: moderate (0), large (1). Character 10 of Smith et al. (2018).
  6. Coracoid: procoracoid process, size: completely reduced (0); developed but moderate size, projecting approximately ¼ the width of the coracoid shaft (1); large, projecting approximately half the width of the coracoid shaft (2); projecting more than ½ the width of the coracoid shaft (3). Ordered. Character 13 of Smith et al. (2018), adapted from character 11 of Mayr (2004).
  7. Coracoid: medial side: flange absent, margin straight (0); flange present, margin convex (1). Character 14 of Smith et al. (2018).
  8. Sternum: lateral trabeculae, posterior tip: extends posteriorly beyond tips of medial trabeculae (0); of same length or slightly shorter than medial trabeculae (1). Character 17 of Smith et al. (2018).
  9. Humerus: size of dorsal supracondylar process: absent or very small nub (0); well-developed but small (1); large, separated from shaft by a small notch (2). Ordered. Character 18 of Smith et al. (2018); similar content but different coding from character 26 of Mayr (2015). Coding for *Z. luberonensis* differs from matrix of Smith et al. (2018) (1→2) based on description and supra-specific coding of Mayr (2008, 2015).
  10. Humerus: direction of dorsal supracondylar process: parallel to shaft of humerus (0); laterally directed in relation to humeral shaft (1). Character 19 of Smith et al. (2018).
  11. Humerus: bicipital crest, size: moderate (0); large (1); exceptionally large, pointed (2). Ordered. Character 20 of Smith et al. (2018).
  12. Humerus:femur ratio: femur longer than humerus (0); femur subequal to humerus (1); femur shorter than humerus (2). Ordered. Character 21 of Smith et al (2018); coding of Oscine + Suboscine SST differs (2/3→1/2).
  13. Humerus: *m. brachialis* origin situated medially (0); laterally (1). Character 6 of Mayr (2008). Character 22 of Smith et al. (2018).
  14. Humerus: humeral head: narrow in posterior view (0); globose (1). Character 23 of Smith et al. (2018).
  15. Humerus: flexor process: short nub extending only slightly distal to the ventral condyle (0); markedly projecting distal to ventral condyle (1). Character 24 of Mayr (2015). Character 24 of Smith et al. (2018).
  16. Ulna:tarsometatarsus ratio: ulna shorter than tarsometatarsus (0); ulna subequal to tarsometatarsus (1); ulna longer than tarsometatarsus (2). Ordered. Modified from character 27 of Smith et al. (2018). Coding based on k-means clustering of Ulna:TMT ratio from *Z*. *grandei*, *Z. luberonensis*, *Z. ochlurus*, *E. americanus*, *P. ballmanni*, *P. danielsi*, *P. longibrachium*, *P. major*, and *P. quintus*, searching for three clusters. This effectively shifts the range for ‘subequal’ Ulna:TMT ratio to 0.9 – 1.0. Codings affected by the change are *Z. grandei*, *P. ballmanni*, and *P. quintus* (0→1).
  17. Carpometacarpus: dorsal margin: protuberance mid-shaft (‘dentiform process’ of Mayr, 1998): absent, dorsal margin straight (0); present, protuberance mid-shaft (1). Protuberance guides major digit tendon of *m. extensor digitorum communis* along dorsal surface of carpometacarpus; highly variable within Coraciimorpha. Character 21 of Mayr (2008); character 46 of Clarke et al. (2009); character 32 of Mayr (2015); character 28 of Smith et al. (2018).
  18. Carpometacarpus: intermetacarpal process: absent (0); present (1). Modified from characters 22 of Mayr (2008), 47 of Clarke et al. (2009), and 33 of Mayr (2015). Character 29 of smith et al. (2018).
  19. Carpometacarpus: intermetacarpal process: if present, unfused to minor metacarpal (0); fused to minor metacarpal (1). Modified from character 47 of Clarke et al. (2009). Character 30 of Smith et al. (2018).
  20. Carpometacarpus: metacarpal III: somewhat curved, concave dorsally (0); straight (1). Character 31 of Smith et al. (2018), modified from character 31 of Mayr (2015). Coding for *E. americanus* differs from matrix of Smith et al. (2018) (0→1) to reflect description from Weidig (2010).
  21. Carpometacarpus: metacarpal III: subequal to metacarpal II (0); longer than metacarpal II (1). Modified from character 24 of Mayr (2004, 2008), character 35 of Mayr (2015). Character 32 of Smith et al. (2018).
  22. Manual digit III, phalanx 1: posterior margin: straight or slightly tapered (0); widened into a small tubercle (1). Noted by Weidig (2010). Character 33 of Smith et al. (2018).
  23. Manual digit I, phalanx 2: absent (0); present (1). Character 34 of Smith et al. (2018).
  24. Manual digit II, phalanx 1: smooth and linear ventrally (0); hooked ventrally (1); convex ventrally (2). Modified from character 56 of Clarke et al (2009). Character 35 of Smith et al. (2018).
  25. Manual digit II, phalanx 2: >50% length of digit II, phalanx 1 (0); ≤50% length (1). Character 63 of Mayr (2015); character 36 of Smith et al. (2018).
  26. Pelvis: obturator foramen: open (0); closed (1). Character 37 of Smith et al. (2018).
  27. Tibiotarsus: cranial cnemial crest, length of anterior projection: less than anteroposterior width of shaft of tibiotarsus (0); subequal (1); greater (2). Ordered. Modified from character 39 of Mayr (2004). Character 38 of Smith et al. (2018).
  28. Tarsometatarsus, anterior end of trochlea metatarsi IV: marked convexity on lateral tarsometatarsal margin: present (0); absent (1). Autapomorphic for *Zygodactylus* (Mayr 2008). Character 39 of Smith et al. (2018).
  29. Tarsometatarsus: marked sulcus on plantar surface of proximal end of trochlea metatarsi IV bordering on lateral tarsometatarsal convexity: absent (0); present (1). Character 40 of Smith et al. (2018); modified from character 42 of Mayr (2008), character 60 of Mayr (2015). Coding of *Z. ignotus* differs from Smith et al. (2018) (0→1) to reflect anatomical description of Ballmann (1969b).
  30. Tarsometatarsus, trochlea metatarsi II: plantarly directed projection: absent (0); present (1). Character 40 of Mayr (2008); character 41 of Smith et al. (2018). Modification from character 56 of Mayr (2015).
  31. Tarsometatarsus: length relative to humerus: <1.0 (0); 1.0 to 1.25 (1); > 1.25 (2). Ordered. Modified from character 42 of Smith et al. (2018), character 34 of Mayr (2004, 2008), character 46 of Mayr (2015). Coding based on k-means clustering of TMT:humerus ratios from *Z*. grandei, *Z. luberonensis*, *Z. ochlurus*, *E. americanus*, *P. ballmanni*, *P. danielsi*, *P. longibrachium*, *P. major*, and *P. quintus*, searching for three clusters.
  32. Tarsometatarsus: plantar surface of metatarsal trochlea: concave (0); linear (1). Noted by Zelenkov (2007). Character 43 of Smith et al. (2018).
  33. Tarsometatarsus: accessory trochlea: absent (0); present (1). Character 69 of Clarke et al. (2009). Character 44 of Smith et al. (2018).
  34. Tarsometatarsus: accessory trochlea (if present): bulbous, reaches at least to middle of trochlea metatarsi III (0); does not reach to trochlea metatarsi III (1). Noted by Mayr (2004). Character 45 of Smith et al. (2018).
  35. Pedal digit I, phalanx 2: less than 1/3 the length of pedal digit II, phalanx 1 (0); longer than 1/3 (1).
  36. Pedal digit III to tarsometatarsus length ratio: ≥85% (0); <85% (1). Character 48 of Smith et al. (2018).
  37. Pedal unguals: degree of curvature: slight to absent (0); moderate (1); pronounced curve (2). Ordered. Character 49 of Smith et al. (2018).
  38. Pedal digit III: ungual to non-ungual phalanges length ratio: <25% (0); ≥25% (1). Character 50 of Smith et al. (2018).
  39. Pedal phalanges: length of digit I, phalanx 1 relative to digit III, phalanx 1: shorter (0); longer (1). Modified from Character 46 of Mayr (2008). State 1 is autapomorphic for *Z. ochlurus* within Zygodactylidae.

# Morphological Character Matrix

|  | 1 | 2 | 3 | 4 | 5 | 6 | 7 | 8 | 9 | 10 | 11 | 12 | 13 | 14 | 15 | 16 | 17 | 18 | 19 | 20 | 21 | 22 | 23 | 24 | 25 | 26 | 27 | 28 | 29 | 30 | 31 | 32 | 33 | 34 | 35 | 36 | 37 | 38 | 39 |
| --- | --- | --- | --- | --- | --- | --- | --- | --- | --- | --- | --- | --- | --- | --- | --- | --- | --- | --- | --- | --- | --- | --- | --- | --- | --- | --- | --- | --- | --- | --- | --- | --- | --- | --- | --- | --- | --- | --- | --- |
| *Zygodactylus ochlurus* n. sp. | ? | ? | ? | 1 | ? | 0 | ? | 1 | 2 | ? | ? | 2 | ? | 0 | 1 | 1 | 1 | 1 | 0 | 1 | 1 | ? | ? | ? | 0 | 0 | 2 | 1 | ? | ? | 1 | ? | 1 | 0 | 1 | 1 | 0 | 0 | 1 |
| *Zygodactylus grandei* | 0 | 0 | 0 | ? | 0 | 2 | 1 | 1 | 2 | 1 | 0 | 2 | ? | 0 | 1 | 1 | 1 | 1 | 0 | 1 | 1 | 1 | 1 | 0 | 1 | ? | 1 | 1 | 1 | 1 | 1 | 0 | 1 | 0 | 1 | 0 | 0 | 1 | 0 |
| *Zygodactylus luberonensis* | ? | ? | 0 | 1 | 0 | 0 | 0 | ? | 2 | 1 | ? | 0 | 1 | 1 | 1 | 0 | 1 | 1 | 0 | 1 | 1 | 0 | 1 | 0 | 0 | ? | 1 | 1 | 1 | 1 | 2 | 0 | ? | 0 | 0 | 0 | 0 | 0 | 0 |
| *Zygodactylus ignotus* | ? | ? | ? | ? | ? | ? | ? | ? | ? | ? | ? | ? | ? | ? | ? | ? | ? | ? | ? | ? | 1 | ? | ? | ? | ? | ? | 1 | ? | 1 | 1 | ? | 0 | 1 | 0 | ? | ? | ? | ? | ? |
| *Eozygodactylus americanus* | 0 | 0 | 0 | ? | 0 | 2 | 1 | 0 | 2 | 1 | 0 | 0 | 1 | 0 | 1 | 0 | 0 | 1 | 0 | 1 | 1 | 1 | 0 | 0 | 1 | 0 | 1 | ? | ? | 1 | 2 | 0 | 1 | 0 | 0 | 0 | 0 | 0 | 0 |
| *Primozygodactylus ballmanni* | 0 | 0 | 0 | ? | ? | 1 | 1 | ? | 1 | 0 | 1 | 1 | 1 | 0 | 1 | 1 | 1 | ? | ? | 1 | ? | 0 | 1 | 0 | ? | 1 | 0 | 0 | 0 | ? | 1 | 0 | 1 | 1 | ? | 1 | 1 | 0 | ? |
| *Primozygodactylus danielsi* | 0 | 0 | 0 | 1 | 0 | 1 | 1 | 1 | 1 | 0 | 1 | 1 | 1 | 0 | 1 | 1 | 1 | 1 | 0 | 0 | 0 | 0 | 1 | 0 | 0 | 1 | 0 | 0 | 0 | 1 | 1 | 0 | 1 | 1 | 1 | 1 | 1 | 0 | 0 |
| *Primozygodactylus eunjooae* | ? | ? | ? | 1 | ? | ? | 1 | ? | ? | ? | ? | ? | 1 | ? | 1 | ? | 1 | ? | ? | ? | ? | 0 | ? | 0 | ? | ? | ? | 0 | 0 | 0 | ? | 0 | 1 | 1 | 1 | 1 | 1 | 0 | ? |
| *Primozygodactylus longibrachium* | ? | ? | ? | 1 | 1 | 1 | 1 | ? | ? | ? | 1 | ? | ? | ? | ? | 2 | ? | ? | ? | ? | ? | ? | ? | ? | 0 | ? | ? | ? | ? | ? | 0 | 0 | 1 | 1 | ? | 1 | 1 | 1 | 0 |
| *Primozygodactylus major* | 0 | 0 | 0 | 1 | 1 | 1 | 1 | 1 | 1 | 0 | 1 | 1 | 1 | 0 | 1 | 2 | 1 | 1 | 0 | ? | ? | 0 | 1 | 0 | ? | 1 | 0 | 0 | 0 | 0 | 0 | 0 | 1 | 1 | ? | 1 | 1 | 0 | ? |
| *Primozygodactylus quintus* | 0 | 0 | 0 | 1 | 1 | 1 | 1 | 1 | 1 | 0 | 1 | 1 | 1 | ? | 1 | 1 | ? | 1 | ? | 1 | 0 | 0 | ? | 0 | 0 | ? | ? | ? | ? | ? | 1 | 0 | 1 | ? | 1 | ? | 1 | 0 | 0 |
| Oscine + Suboscine SST | 0&1 | 0&1 | 0 | 1 | 1 | 0 | 0 | 0 | 1&2 | 0&1 | 1 | 1&2 | 1 | 0&1 | 1 | 1&2 | 1 | 1 | 1 | 0 | ? | 0 | 0 | 0&2 | 1 | 1 | 2 | 0 | 0 | 0 | 0&1 | 1 | 0 | ? | ? | 1 | 1&2 | 1 | 1 |
| *Acanthisitta chloris* | 0 | 0 | 1 | 1 | ? | 1 | ? | ? | 1 | 1 | 0 | 2 | 1 | ? | 1 | 0 | ? | 1 | 1 | 1 | ? | 0&1 | 0 | ? | ? | 1 | 2 | 0 | 0 | 0 | 2 | 1 | 0 | ? | ? | 1 | 2 | 0 | 1 |
| *Jamna szybiaki* | 0 | 0 | 1 | ? | ? | ? | 0 | ? | 2 | 0 | 0 | 2 | ? | ? | 1 | ? | ? | 1 | 1 | 0 | 1 | 0 | 0 | ? | 0 | ? | 0 | ? | ? | ? | ? | ? | ? | ? | ? | ? | ? | ? | ? |
| *Nestor* SST | 1 | 1 | 0 | 0 | 1 | 3 | 0 | ? | 0 | ? | 2 | 1 | 0 | 0 | 0 | 0 | 0 | 0 | ? | 1 | ? | 0 | 0 | 2 | ? | 1 | 1 | 0 | 0 | 1 | 0 | 0 | 1 | 0 | ? | ? | 2 | ? | 0 |
| *Cyrilavis colburnorum* | 1 | 1 | 1 | 0 | 0 | 3 | 1 | 1 | 0 | ? | 1 | 2 | 0 | 0 | 0 | 2 | 0 | 0 | ? | 1 | 0 | 0 | 0 | ? | 0 | ? | 0 | ? | ? | 1 | 0 | 0 | 1 | 1 | ? | 1 | 2 | 1 | 1 |
| Piciform SST | 1 | 1 | 1 | 1 | 1 | 1 | 0 | 1 | 1 | 1 | 1 | 2 | 1 | 0 | 1 | 1 | 0 | 1 | 1 | 0 | ? | 0 | 0 | 0&1 | ? | 1 | 0 | 0 | 0 | 1 | 0 | 0 | 1 | 0 | ? | 1 | 1&2 | 1 | 0 |
